# Properties of Concrete Made with Low-Emission Cements CEM II/C-M and CEM VI

**DOI:** 10.3390/ma13102257

**Published:** 2020-05-14

**Authors:** Anna Król, Zbigniew Giergiczny, Justyna Kuterasińska-Warwas

**Affiliations:** 1 Faculty of Mechanical Engineering, Opole University of Technology, Prószkowska Str. 76, 45-758 Opole, Poland; 2 Faculty of Civil Engineering, Silesian University of Technology, Akademicka Str. 5, 44-100 Gliwice, Poland; zbigniew.giergiczny@polsl.pl; 3 Institute of Ceramics and Building Materials, Oświęcimska Str. 21, 45-641 Opole, Poland; j.kuterasinska@icimb.pl

**Keywords:** ternary cements, limestone, siliceous fly ash, granulated blast furnace slag, concrete properties, concrete durability, CO_2_ emission

## Abstract

The paper presents the composition and properties of low-emission ternary cements: Portland multicomponent cement CEM II/C-M and multicomponent cement CEM VI. In the ternary cements, Portland clinker was replaced at the levels of 40% and 55% with a mixture of the main components such as limestone (LL), granulated blast furnace slag (S) and siliceous fly ash (V). Portland multicomponent cements CEM II/C-M and CEM VI are low-emission binders with CO_2_ emissions ranging from 340 (CEM VI) kg to 453 (CEM II/C-M) kg per Mg of cement. The results obtained indicate the possibility of a wider use of ground limestone (LL) in cement composition. This is important in the case of limited market availability of fly ash and granulated blast furnace slag. The tests conducted on concrete have shown that the necessary condition for obtaining a high strength class and durability of concrete from CEM II/C-M and CEM VI ternary cements is low water–cement ratio. Durability characteristics of concrete (carbonation susceptibility, chloride ion permeation, frost resistance) made of CEM II/C-M and CEM VI cements were determined after 90 days of hardening. This period of curing reflects the performance properties of the concrete in a more effective way.

## 1. Introduction

In 2017, the global production of cement, the base component of concrete, amounted to almost 4.65 billion Mg [1]. For the production of 1 Mg of Portland cement clinker, about 1.7 Mg of natural resources are used, mainly carbonate raw materials such as limestone and marl. Thus, as a result of the clinker firing process, huge amounts of CO_2_ are released into the atmosphere, the source of which is the thermal dissociation of carbonates in the raw material bulk (60%) and the emission of CO_2_ from the combustion of technological fuel (40%) [2,3]. It is considered that cement production is responsible for about 7.4% of the world carbon dioxide emission (2.9 Mg in 2016) [4]. Therefore, the world cement industry has to meet the constantly growing environmental requirements, which mainly concern the reduction of dust and greenhouse gas emissions [5]. Unfortunately, the production of the basic component of cement, i.e., Portland clinker, is associated with the emission of CO_2_, which is about 825–890 kg of CO_2_ per Mg of clinker [6]. The world average is about 840 kg of CO_2_ but the carbon dioxide emission level should be lower than 400 kg per Mg of cement. It is suggested that the emission levels reach around 350–410 kg per Mg of cement [4].

The possibilities of emission reduction include two solutions in the cement production process [2,7]:

- production of multicomponent cements CEM II-CEMV according to EN 197-1 [8] using significant quantities of main ingredients other than Portland clinker;
- modification of the production process of cement clinker by modification of the raw material set (belite clinkers, belite-sulphate-aluminate clinkers, etc.) and use of alternative (non-fossil) biomass-rich fuels.

In the case of production of CEM II-CEM V multicomponent cements, the main components are usually by-products of industrial processes such as siliceous fly ash (V), calcareous fly ash from coal dust combustion in the power industry or granulated blast furnace slag from iron metallurgy (S) [2,9,10]. The cements containing significant amounts of fly ashes and slags are characterized by low hydration heat (a feature important in the implementation of massive concrete structures), higher strength after longer curing periods and higher resistance to chemical aggression [2,9,10,11]. To ensure appropriate durability of concrete made of cement with lower clinker content in the assumed construction environment, the concrete composition (type and amount of cement, w/c ratio, type of admixtures and amount of concrete additives) should be properly designed, so that the concrete is characterized by a tight matrix. Determining the concrete tightness, e.g., by limiting the amount of water in the concrete mix or using cement with mineral additives, results in limiting the capillary porosity of the hardened cement slurry [2,12,13,14]. On the “macro” scale, it directly affects the depth of penetration of aggressive media and the size of capillary pull, whereas on the “micro” scale, it results in impeding the diffusion of aggressive ions into the cement matrix. However, the availability of fly ash and granulated blast furnace slag, with increasing cement production, is limited [15]; therefore, limestone (LL) is used increasingly often in cement composition. The main advantage of this component is its widespread availability and the fact that it can be obtained from the cement plants own raw material resources [16,17,18].

Calcium carbonate, the main component of limestone, reacts with calcium aluminates to form hydrated calcium carboaluminates. The presence of hydrated calcium carboaluminates inhibits the transition of ettringite to monosulfate, thus, in hydration products the amount of monosulfate decreases or disappears while the amount of ettringite increases [19]. The fact that calcite reacts with C_3_A to form carboaluminates means that CaCO_3_ may play, to a limited extent, the role of a regulator of setting time. This results in the reduction of the amount of gypsum, which is necessary to regulate the setting time [20].

In addition to the reaction with calcium aluminate, the addition of limestone to the cement may accelerate the C_3_S phase reaction. This effect is explained by the nucleation effect, in which CaCO_3_ grains act as additional crystallization germs for cement hydration products [19,20,21]. Limestone is a very soft component in comparison to Portland clinker. After the milling process, it has a much higher specific surface area and, as a micro-filler, influences the properties of cement composites, e.g., by reducing porosity, increasing strength in the initial period of hardening and improving workability, reducing water consumption and reducing water draining from the concrete mixture (so-called “bleeding”) [19,20,21,22,23,24]. Bearing these facts in mind, the European Committee for Standardization CEN has undertaken standardization works aimed at extending the range of cements containing cement components other than Portland clinker in its composition. It is proposed to implement the non-harmonized standard prEN 197-5 [25], which extends the range of Portland multicomponent cements (the possibility of using several main components in the composition of cement) by a group of Portland multicomponent cements CEM II/C-M with a minimum content of Portland clinker of 50% and a newly created group of multicomponent cements CEM VI, in which the share of non-clinker components may be a maximum 65%.

This paper presents the results of research on Portland multicomponent cement CEM II/C-M with 40% of non-clinker main components and multicomponent cement CEM VI with 55% of these components. Ground granulated blast furnace slag (S), siliceous fly ash (V) and ground limestone (LL) were used as non-clinker main components. Concrete tests were performed for the analyzed cements CEM II/C-M and CEM VI. The basic properties of concrete mixture and hardened concrete were determined with a view to future use of cements in construction practice. The level of CO_2_ emissions originating in the composition of CEM II/C-M and CEM VI cement was also calculated, as well as the level of CO_2_ emissions from the production of concrete with the use of tested cements.

## 2. Materials and Methods

### 2.1. Characteristics of Components and Composition of Tested Cements

Three types of non-clinker ingredient were used in the study: granulated blast furnace slag from iron metallurgy, siliceous fly ash from the combustion of coal in power plants, and natural limestone.

**Figure 1:**
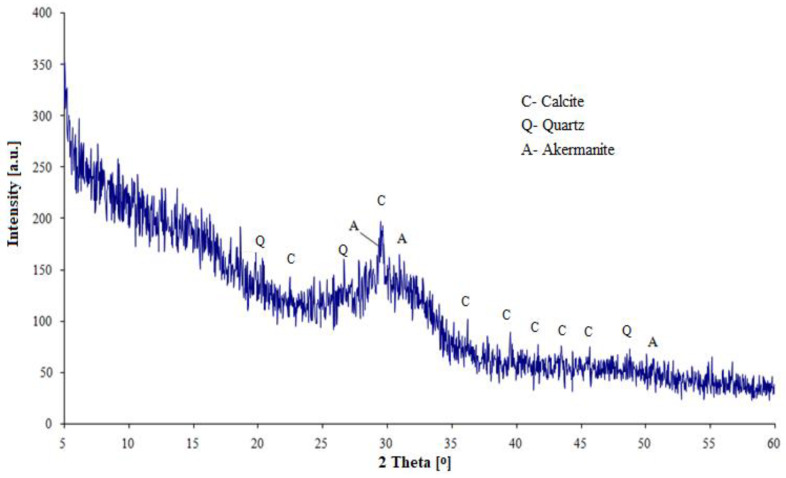
Diffractogram of granulated blast furnace slag.

**Figure 2:**
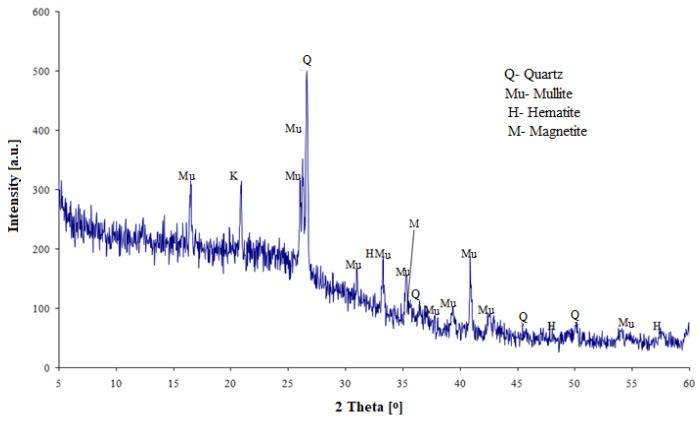
Diffractogram of siliceous fly ash.

The chemical composition of the cement components and selected physical properties are given in Table 1. Figure 1 and Figure 2 show diffractograms of ground granulated blast furnace slag (S) and fly ash (V).

In the slag phase composition (S), the dominant component is the vitreous phase, the quantitative content of which (determined microscopically) is 98%. In fly ash, next to the vitreous phase, the main crystalline components identified are quartz, mullite, hematite and magnetite. The Portland cement CEM I is a semi-finished product with an increased SO_3_ content (5.0%) in order to obtain a normal SO_3_ content (max. 3.5%) when mixed with the other main components of the cement. Therefore, this cement is a semi-finished product in the process of manufacturing multicomponent cements. The clinker content in the cement was 90%.

**Figure 3:**
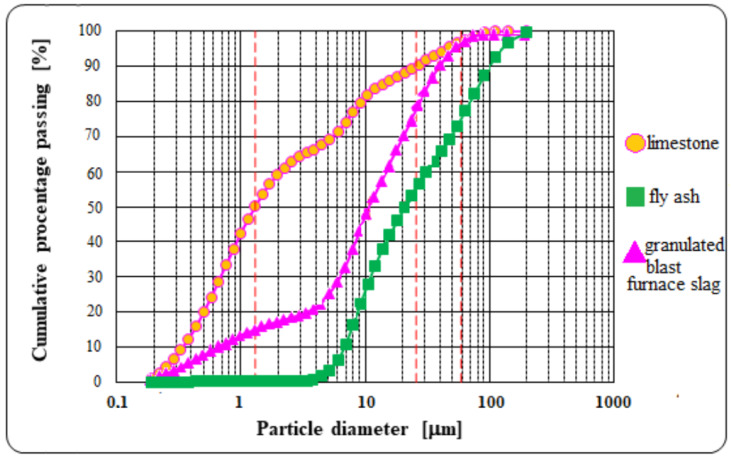
Particle size distribution of supplementary cementitious materials.

When analyzing the properties of the main components of cement used, attention should be paid to the high specific surface area of limestone of 6150 cm^2^/g (Table 1). Obtaining such a high specific surface area is relatively easy due to the very good granularity of the limestone. The granulometric composition of non-clinker cement components is shown in Figure 3.

Two CEM II/C-M cements with a Portland clinker content of 54% and two CEM VI cements with a Portland clinker content of 40.5% were prepared for testing the ternary cements. The composition of the tested cements and CO_2_ emission levels are given in Table 2. The CO_2_ emission level from 1 Mg of cement was calculated assuming the average CO_2_ emission level of production of 1 Mg of clinker at the level of 840 kg [4] and the clinker content in the composition of the tested ternary cements CEM II/C-M and CEM VI (Table 2). In the calculations, the CO_2_ emission level related to the transport and grinding of the components into cement was omitted. The obtained CO_2_ emission levels from 1 Mg of tested cements at the level of 340.2–453.6 kg allows the inclusion of CEM II/C-M and CEM VI cements into low-emission cements.

In order to evaluate the synergy effects of cement components, comparative cements containing one non-clinker component—slag cement C(55S), fly ash cement C(40V) and limestone cement C(40LL) were also tested (Table 2).

The properties of cements were determined according to the procedures of EN 196 and the density according to the standard on the properties of aggregates EN 1097-7 (Table 3).

### 2.2. Concrete Composition and Properties’ Test Methods

Based on Portland multicomponent cements CEM II/C-M and multicomponent cements CEM VI, concrete mixes with the following composition, given in Table 4, were designed.

Two types of concrete were prepared—type I containing 300 kg of cement in 1 m^3^ of concrete mix at a ratio w/c = 0.60 and type II, containing 340 kg of cement in 1 m^3^ of concrete mix at a ratio w/c = 0.35. Natural gravel aggregate with a fraction up to 16 mm and sand 0–2 mm as fine aggregate were used in the concrete mixture. A superplasticizer (PCE) based on polycarboxylate ether was used in the composition of concrete with reduced water–cement ratio (w/c = 0.35).

The properties of concrete mixtures and hardened concrete were tested according to the methodology included in the standards, which are presented in Table 5.

## 3. Results and Discussion

### 3.1. Properties of CEM II/C-M and CEM VI Cements

The properties of the cements are presented in Table 6. The density of the cements was lowest for those containing siliceous fly ash (Table 6). The specific surface area of ternary cements ranged from 4350 to 4750 cm^2^/g. Cement slurries with ternary cements did not show volume changes due to swelling (Table 6). The setting time of cements and other properties are closely related to their composition and the amount of mineral additives introduced (Table 6).

**Figure 4:**
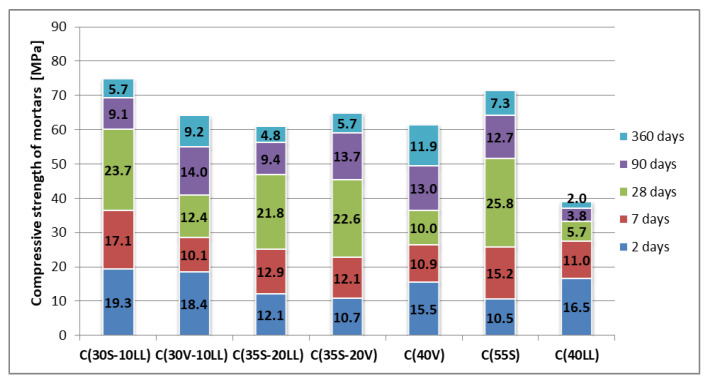
Increase in compressive strength depending on the curing time of cement mortars.

In most cases, the highest increases in strength of standard cement mortars can be observed between 7 and 28 days of curing (Figure 4). During this period, the strength increases quite significantly in the case of cements containing blast furnace slag (the slag is hydraulically active and begins to react with water a long time before the ash pozzolanic reaction begins). The highest strength increase in this period is observed in comparative slag cement C(55S) on Figure 4. Partial replacement of blast furnace slag, both with limestone LL and fly ash V, slightly reduces the strength increase between the 7^th^ and 28^th^ days of hardening. Omitting the small influence of limestone on the increase in early strength, it is a rather chemically inert component in the cement system, and therefore its addition to slag causes a decrease in later strength. Replacement of slag with fly ash also slows down the dynamics of strength growth. This can be explained by the fact that the pozzolanic reaction of fly ash begins intensify only after 28 days of curing (when the amount of Ca(OH)_2_ from cement hydration increases). The final strength (after 360 days) of multicomponent cement C(30S-10LL) is similar to that of comparative cement C(55S), while in the case of cement C(35S-20V) there is a slight decrease in the final strength, compared to cement C(55S) (Figure 4). This decrease can be explained by the lower activity of fly ash in relation to ground granulated blast furnace slag, which is a component with latent hydraulic activity (with a CaO content of approximately 40–44% and after heat treatment in a blast furnace under conditions similar to those in a rotary kiln for Portland clinker production).

The partial replacement of fly ash by limestone C(30V-10LL) in cement composition, allows a cement to be made with higher early strength (after 2 days) and higher final strength than the reference cements C(40V) and C(40LL). The higher early strength in the presence of limestone may result from the caulking effect as well as the small amount of carboaluminates formed. On the basis of the results obtained, it can also be observed that the addition of LL limestone decreases the strength of cement mortars to a greater extent in combination with siliceous fly ash (C(30V-10LL)), while the use of limestone (even up to 20%) in combination with ground granulated blast furnace slag (cements: C(30S-10LL), C(35S-20LL)) gives a much smaller decrease, slightly less than in the case of using the S slag composition with fly ash V-cement C(35S-20V).

### 3.2. Concrete Properties with CEM II/C-M and CEM VI Low-Emission Cements

#### 3.2.1. Properties of Concrete Mixture

The properties of concrete mixture are presented in Table 7. The consistency (fall cone method) of concrete mixtures at w/c = 0.60 corresponded to class S3 for concrete mixture with cement C(30S-10LL) and classes S2 for other cements. Reduction of the water content to the level w/c = 0.35 caused a decrease in consistency to class S1. 

The density of all concrete mixtures with the ratio w/c = 0.60 was similar and ranged from 2350 to 2360 kg/m^3^. The air content was low and ranged from 0.6% to 1.0%. Decreasing the water-cement ratio to the level w/c = 0.35 resulted in an increase in the density of concrete mixtures by about 70–80 kg/m^3^ and an increase in air content to 1.6–2%. The increase in aeration of the concrete mixture is typical of using liquefying admixture.

#### 3.2.2. Properties of Hardened Concrete

- Compressive strength

**Figure 5:**
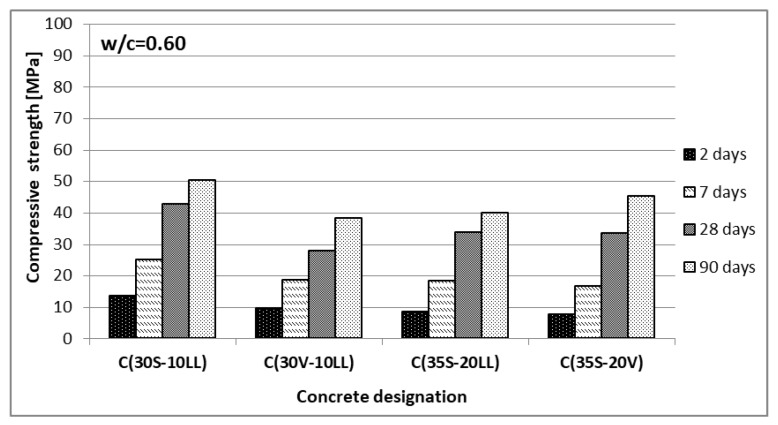
Compressive strength of concrete (w/c = 0.60).

**Figure 6:**
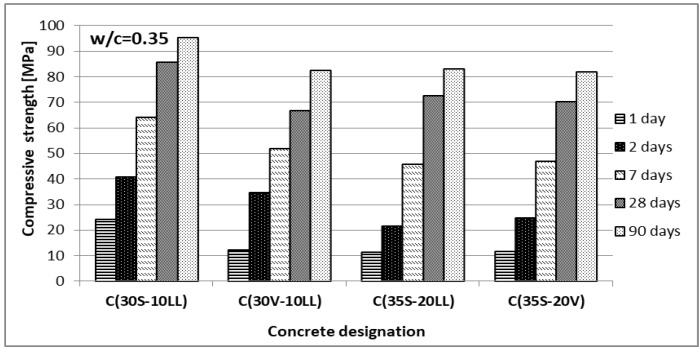
Compressive strength of concrete (w/c = 0.35).

The compressive strength was determined, after 2, 7, 28 and 90 days of curing, on 10 cm cubic samples. For concretes with w/c = 0.35, strength tests were also performed after 1 day of curing. The results are presented in Figure 5 and Figure 6.

The highest compressive strength, at w/c = 0.35 and 0.60, was obtained by concrete made of Portland multicomponent cement C(30S-10LL). The lowest compressive strength was achieved with C(30V-10LL) on Portland ash and lime cement, despite the fact that it contained more Portland clinker compared to the other two CEM VI cement concretes. Limestone and siliceous fly ash show a synergistic effect only with early strength (2 days) and only with 10% addition of limestone, which confirms the results obtained by De Werdt et al. [40,41,42]. The results obtained from strength tests of concretes made of slag-calcareous cements provide a reason for the prospective wider use of limestone in cement composition. 

**Figure 7:**
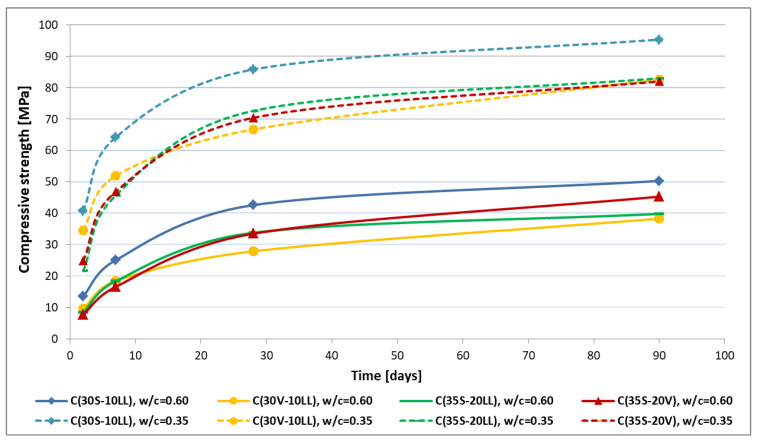
The impact of reduced w/c ratio on the compressive strength of concretes made of tested ternary cements.

Decreasing the water-cement ratio (w/c) from 0.60 to 0.35 resulted in a significant increase in the compressive strength of concretes made of all cements tested (Figure 7). This increase is particularly visible in the initial period of concrete hardening, i.e., until the 7^th^ day. It can be noted that the lowest results were obtained for concrete using cement C(30V-10LL), however, the compressive strength after 28 days was nearly 70 Mpa and was almost twice as high as the strength obtained at w/c = 0.60. To sum up, it should be stated that a low w/c ratio is a very effective factor in shaping the strength characteristics of concrete made of cements with low Portland clinker content (Figure 7).

- Water absorption and water penetration under pressure

**Figure 8:**
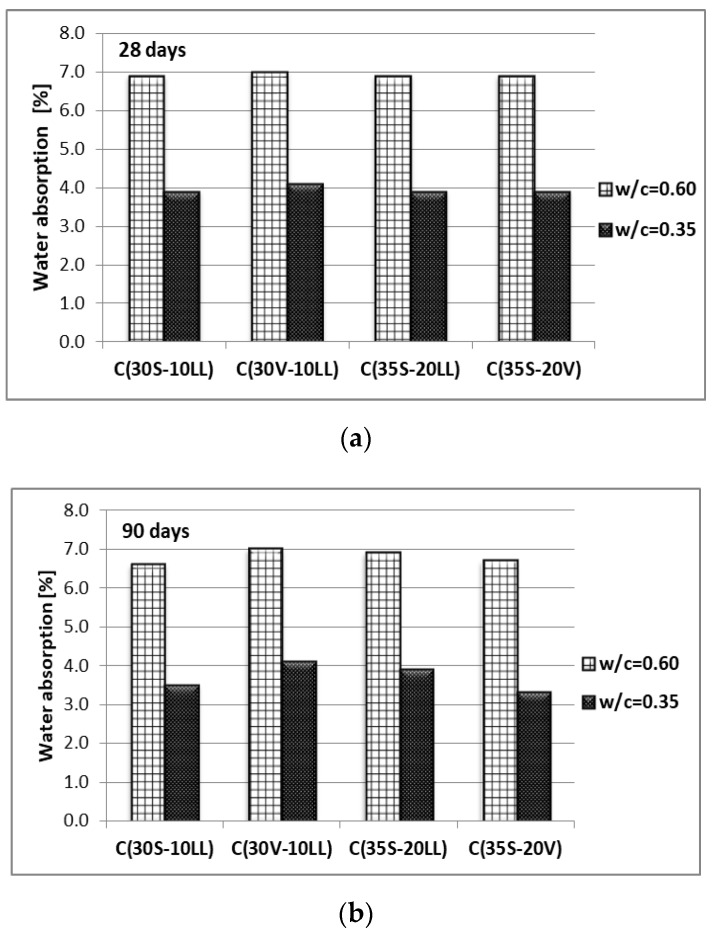
Water absorption of concrete after: (**a**) 28 days of curing, (**b**) 90 days of curing.

All tested concretes, at the same w/c, show similar water absorption. For concretes with w/c = 0.60 the absorption varies between 6.6% to 7.0%, while for concretes with reduced w/c = 0.35 it is much lower and ranges from 3.3% to 4.1% (Figure 8). Extension of the curing time to 90 days resulted in a slight decrease in absorption of the tested concretes, most noticeable for concrete made with C(35S-20V) cement.

**Figure 9:**
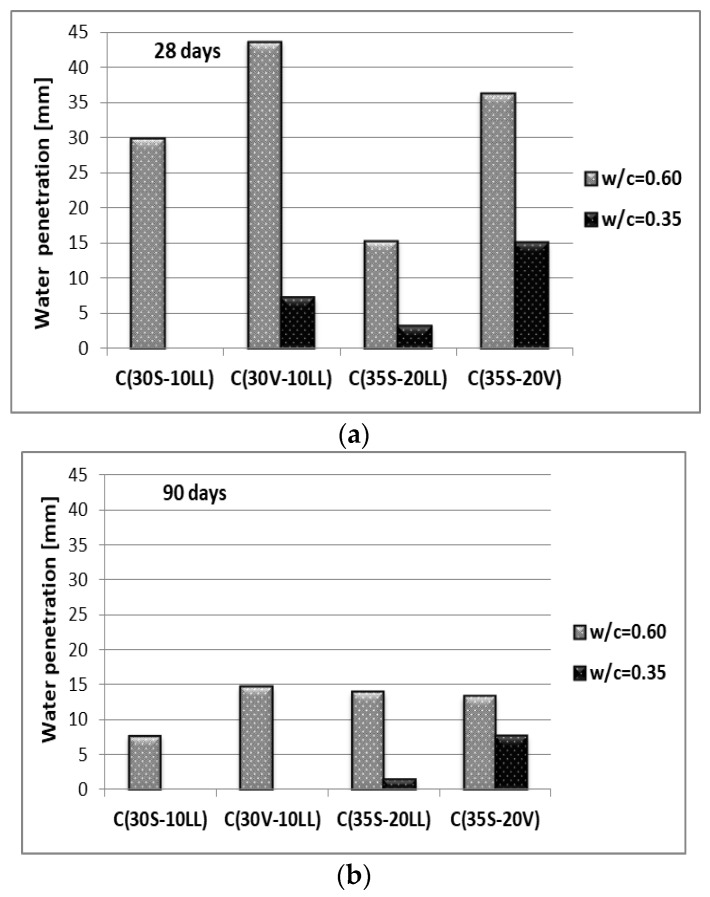
Water penetration depth of concrete after: (**a**) 28 days of curing, (**b**) 90 days of curing

The results of the study on the depth of water penetration under pressure (Figure 9) show that concretes with w/c = 0.35 are characterized by very high tightness, especially after 90 days of curing. The depth of water penetration under pressure at w/c = 0.35 was maximum 15 mm for concrete with cement C(35S-20V) after 28 days of curing. Concretes with w/c = 0.60 showed water penetration depth after 28 days of hardening at the level from 15.3 mm to 43.7 mm and from 7.7 mm to 14.7 mm for concrete curing for 90 days (Figure 9).

Omitting the influence of the w/c ratio, the differences in the depth of water penetration inside the concrete matrices primarily result from the different activity of the main components of cements used. The most active component, apart from Portland clinker, is ground granulated blast furnace slag, whereas fly ash is a component with pozzolanic activity (ability to react in the presence of moisture, with Ca(OH)_2_ from the hydration of silicate phases of Portland clinker). The impact of this reaction on the properties of mortar (concrete) is earliest visible about 28 days and later (Figure 9b). The addition of limestone improves the porosity of the cement-ash/slag system. Limestone, as a soft component, is ground into very fine grains, which fill the voids between cement and ash/slag grains. It results in increased early strength (after 2 days) in relation to the cement included only fly ash. After a longer period of time (28 days and later), cements containing granular blast furnace slag in the composition with ash (S, V) or limestone (S, LL) have higher strength and tightness.

- Carbonation susceptibility

**Figure 10:**
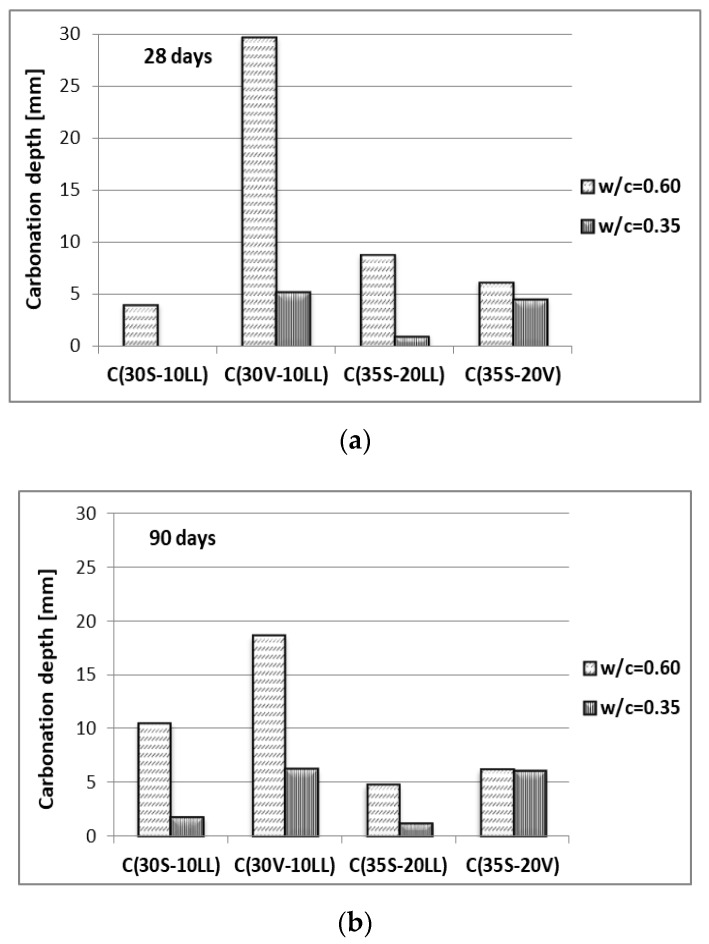
Carbonation depth of concrete after: (**a**) 28 days of curing, (**b**) 90 days of curing.

The type of cement used was assessed for its susceptibility to carbonation (Figure 10). The test was carried out using an elevated CO_2_ concentration 4%, the test duration was 70 days (accelerated method). Analyzing the results obtained for concretes at w/c = 0.60, it can be seen that the highest depth of carbonation is characterized by concrete with Portland multicomponent cement C(30V-10LL), after 28 days of hardening the depth of carbonation reaches 29.7 mm, and after 90 days it is 18.6 mm. Reducing the water–cement ratio to the level w/c = 0.35 very effectively lowered the depth of concrete carbonation (Figure 10). A significant decrease in the depth of carbonation linked with the extension of curing period should be associated with the activity of the cement components used, mainly ground granulated blast furnace slag (hydraulic activity) and fly ash (pozzolanic activity). Additional amounts of products formed later (after 90 days of curing) from the course of reaction between cement hydration products and active mineral additives, settle in the pores of hardening cement slurry and hinder the permeation and penetration of aggressive ions [21,23].

- Chloride ions permeation

**Figure 11:**
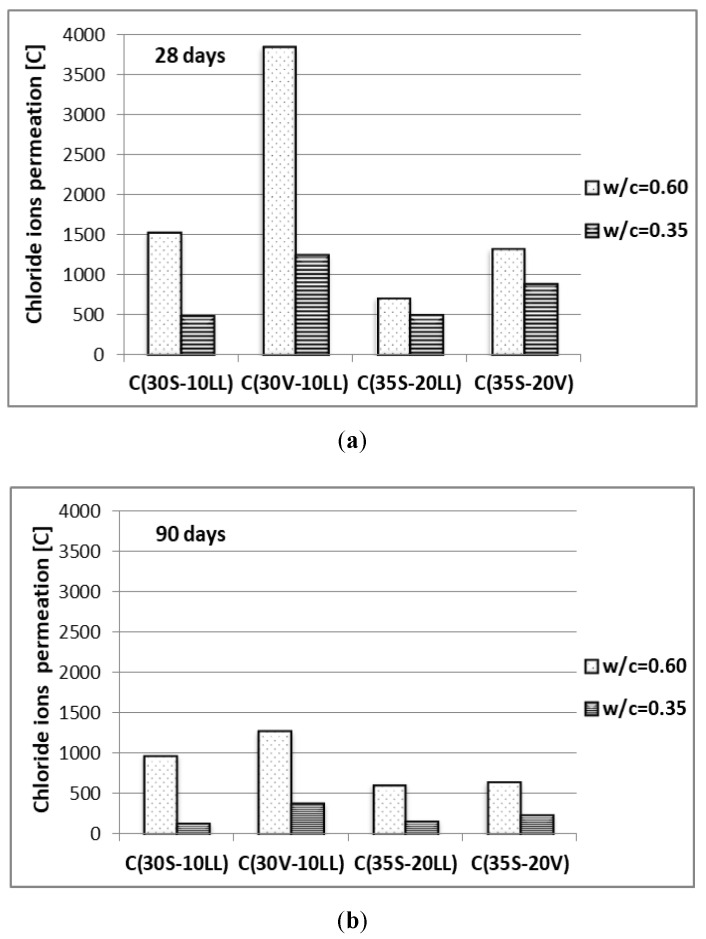
Chloride ions permeation of concrete after: (**a**) 28 days of curing, (**b**) 90 days of curing.

The permeability limitation of the concrete matrix is confirmed by the results of chloride ion permeation (Figure 11). Extending the curing period to 90 days or decreasing the water and cement ratio to w/c = 0.35 results in a significant decrease in the permeation of chloride ions corresponding to low or very low permeation class according to ASTM C 1202-05 [37] for both test dates. The differences in chloride ion permeability between concrete samples can be explained in the same way as was described in the water penetration analysis, as this feature is strictly related to tightness of concrete.

- Freeze–thaw resistance

**Figure 12:**
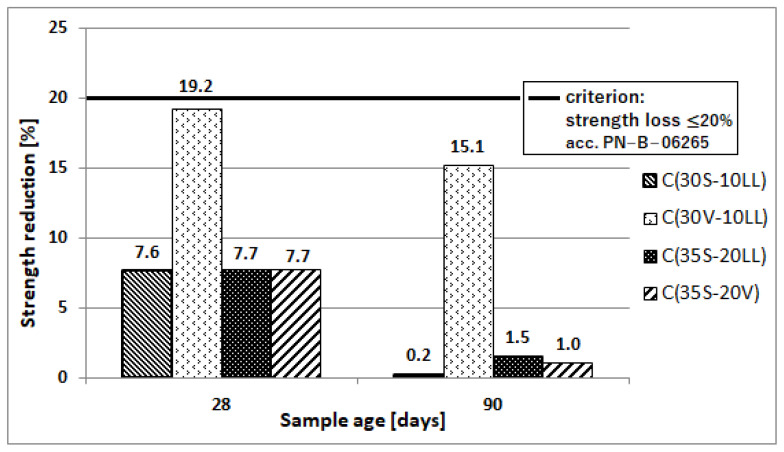
Frost resistance of concretes tested by the standard method [38].

**Figure 13:**
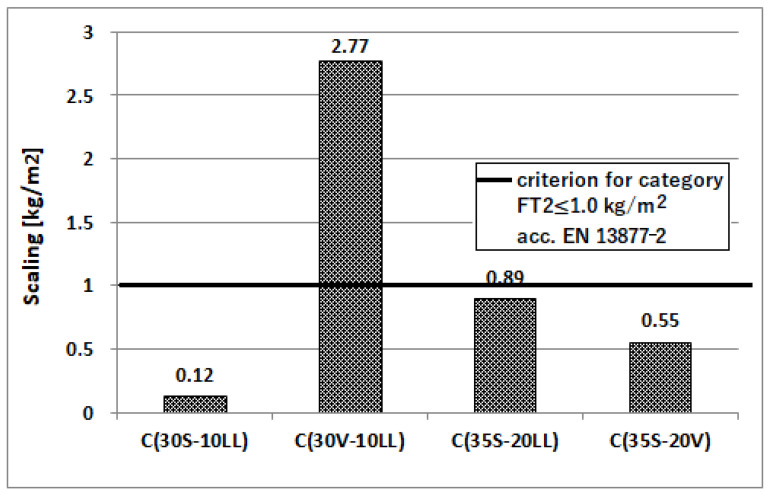
Frost resistance of concretes in the presence of de-icing salt in relation to resistance category according to EN 13877-2:2013-08 [43].

An important feature of concrete, used in areas with minus temperatures, is its resistance to such environmental impact. Resistance of concrete to cyclic freezing and unfreezing was determined by the ordinary method (150 cycles of freezing at −18 °C and unfreezing of concrete at 18 °C, duration time of 1 cycle was 6 h (3 h of freezing and 3 h of thawing)) for concretes with coefficient w/c = 0.35. The test was performed after 28 and 90 days of concrete curing. The result of the test is positive if the decrease of compressive strength is less than 20% and the loss of mass is not more than 5% of weight. The test of concrete surface resistance to frost (56 cycles) in the presence of NaCl de-icing salt was also performed. Concrete was evaluated after 28 days of curing. The test results are presented in Table 8 and Figure 12 and Figure 13.

The type of applied cement affects the durability of concrete under cyclic freezing and unfreezing conditions, especially when de-icing agents are used. The worst results were obtained for concrete made of Portland multicomponent cement C(30V-10LL) (Figure 12 and Figure 13; Table 8). The decrease in compressive strength after the frost resistance test using the normal method reached 19.2% for concrete subjected to alternating temperatures after 28 days of curing and 15.1% for concrete subjected to the test after 90 days of curing. For comparison, concretes made of other cements were characterized by strength decreases at a much lower level of 7.6–7.7% for 28-day samples and 0.2–1.5% for 90-day samples. Concrete samples after the frost-resistance test did not show significant changes in mass.

When analyzing the results of the surface resistance of concrete to frost in the presence of de-icing salt (Table 8, Figure 13), it is clear that only concrete made of Portland multicomponent cement C(30S-10LL) can be classified as resistant. However, remaining concretes, especially concrete made of Portland multicomponent cement C(30V-10LL), show considerable scaling.

#### 3.2.3. CO_2_ Emissions from Concrete Made of CEM II/C-M and CEM VI Cement

The concrete emission level was calculated based on the level of CO_2_ emissions from 1 Mg of cement (Table 2). The results are presented in Table 9. The obtained CO_2_ emission levels per 1 m^3^ of concrete are very low (Table 2). When converting the level of CO_2_ emission into 1 MPa of compressive strength, it can be observed that an important factor is the w/c ratio in concrete. With a lower w/c ratio (0.35), the strength levels are much higher and the difference in the level of the obtained compressive strength depending on the composition of the cement starts to fade. The CO_2_ emission level per 1 MPa is significantly reduced also, e.g., in the case of CEM II/C-M (30V-10LL) cement from 4.9 kg (w/c = 0.60) to 2.3 kg (w/c = 0.35). Considering the compressive strength after 90 days, CO_2_ emissions are lower, on average by about 0.7 kg at w/c = 0.60 and about 0.2 kg at w/c = 0.35. This is due to the fact that cements with a high content of the main components other than Portland clinker (mainly granulated blast furnace slag and/or fly ash) have a significant strength increment between 28 and 90 days of hardening.

## 4. Conclusions

Based on the results of the research carried out, the following conclusions were drawn:

1. The results of the research as well as ecological aspects (mainly reduction of CO_2_ emissions) confirmed the advisability of further development of the assortment of general-use cements with the following ternary cements: Portland multicomponent cement CEM II/C-M and multicomponent cement CEM VI. The properties of these cements and the concretes made with them are the result of the properties of the main components and the synergistic interaction of the composition: granulated blast furnace slag S-limestone LL, siliceous fly ash V-limestone LL, granulated blast furnace slag S-siliceous fly ash V.
2. When evaluating the synergy effect of additives in the composition of cements, it can be observed that much better strength effects are obtained in the composition of ground granulated blast furnace slag (S) with limestone (LL) than fly ash (V) with limestone (LL). This is due to the higher activity of the ground slag compared to fly ash (V). The results obtained also confirm a very good synergy effect, known from construction literature and practice, between slag (S) and fly ash (V). A characteristic feature of CEM II/C-M and CEM VI ternary cements is a low level of early compressive strength (up to 7 days) and a significant increase in compressive strength at later stages (especially between 28 and 90 days of hardening).
3. A typical attribute of CEM II/C-M and CEM VI ternary cements is the low level of early compressive strength (up to 7 days) and a significant increase in compressive strength at a later date (especially between 28 and 90 days of hardening).
4. The results obtained indicate the possibility of wider use of ground limestone LL in cement (concrete) composition. This is important in terms of the limited market availability of fly ash and granulated blast furnace slag.
5. The results obtained from tests on concrete showed that a low w/c ratio is a prerequisite for obtaining high strength class and durability of concrete made of CEM II/C-M and CEM VI ternary cements. This can be achieved by using the latest generation of liquefying admixtures (superplasticizers).
6. The durability characteristics of concrete (carbonation susceptibility, chloride ion permeation, frost resistance) from CEM II/C-M and CEM VI cements should be determined after 90 days of hardening. This period of curing better reflects the performance properties of the concrete.
7. Portland multicomponent cements CEM II/C-M and CEM VI are low-emission binders with CO_2_ emissions ranging from 340 (CEM VI) kg to 453 (CEM II/C-M) kg per Mg of cement. Because of a high content of non-clinker main components, these cements characterize a much lower degree of hydration after 28 days of hardening than ordinary Portland cement CEM I. The presence of slag (S) and fly ash (V) results in a significant increase in the strength of low-emission cements between 28 and 90 days of hardening, and hence a better effect in terms of reducing emissions per 1 MPa is achieved. In some countries, Poland among others, for this reason the quality of concrete with low-emission cements is assessed after 90 days.

## Figures and Tables

**Table 1:** Chemical composition and physical properties of the main components of cement.

Type of Raw Material	Content of Component, (wt. %)									Specific Gravity (g/cm^3^)	Specific Area ACC. Blaine (cm^2^/g)
	SiO_2_	Al_2_O_3_	Fe_2_O_3_	CaO	MgO	SO_3_	Na_2_O	K_2_O	Cl^−^		
Limestone (LL) ^(1)^	5.4	1.3	1.1	49.7	1.8	0.03	<0.1	<0.1	0.005	2.70	6150
Granulated blast furnace slag (S)	40.5	7.4	1.26	43.7	5.0	0.14	0.77	0.45	0.046	2.92	3800
Siliceous fly ash (V) ^(2)^	52.3	27.5	5.80	3.6	2.6	0.29	0.94	3.15	0.008	2.14	2750
Portland cement CEM I	20.65	5.1	2.57	62.94	1.4	5.0	0.15	0.63	0.07	3.16	4500

^(1)^ CaCO_3_ content calculated on the basis of the amount of CaO is 89 (wt.%), total organic carbon (TOC)-0.04 (wt.%), clay content-0.4 g/100 g, ^(2)^ LOI of siliceous fly ash V-2.43 (wt.%) (Category A according to EN 197-1:2012 [8]).

**Table 2:** Composition of tested cements and CO_2_ emission level.

Cement Marking	Type of Cement	Content of Component, (wt. %)				CO_2_ Emission Level from Mg of Cement, (kg)
		Cement CEM I	Slag S	Limestone LL	Fly Ash V	
C(30S-10LL)	CEM II/C-M (30S-10LL)	60	30	10	-	453.6
C(30V-10LL)	CEM II/C-M (30V-10LL)	60	-	10	30	453.6
C(35S-20LL)	CEM VI (35S-20LL)	45	35	20	-	340.2
C(35S-20V)	CEM VI (35S-20V)	45	35	-	20	340.2
C(40V) ^(1)^	CEM (40V)	60	-	-	40	453.6
C(55S) ^(1)^	CEM (55S)	45	55	-	-	340.2
C(40LL) ^(1)^	CEM (40LL)	60	-	40	-	453.6

^(1)^ comparative cements.

**Table 3:** Procedures used to determine the properties of cement.

Property	Standard Test Method
Constancy of volume	EN 196-3:2016-11 [26]
Initial setting time	EN 196-3:2016-11 [26]
Specific surface area	EN 196-6:2011 [27]
Density	EN 1097-7:2008 [28]
Compressive strength	EN 196-1:2016-05 [29]

**Table 4:** Mix proportions of concrete mixtures.

Type	Concrete Designation	w/c	Cement Content (kg/m^3^)	Water (kg/m^3^)	PCE Admixture (kg/m^3^)	Coarse Aggregate (kg/m^3^)		Sand 0–2 mm (kg/m^3^)
						8–16 mm	2–8 mm	
I	C(30S-10LL)	0.60	300	180	-	680	530	680
	C(30V-10LL)		300	180	-	680	530	680
	C(35S-20LL)		300	180	-	680	530	680
	C(35S-20V)		300	180	-	680	530	680
II	C(30S-10LL)	0.35	340	120	10.2	725	565	725
	C(30V-10LL)		340	120	10.2	725	565	725
	C(35S-20LL)		340	120	10.2	725	565	725
	C(35S-20V)		340	120	10.2	725	565	725

**Table 5:** Procedures used to determine the properties of the concrete mixture and hardened concrete.

Property	Standard Test Method (Procedures)
Consistency (fall cone method)	EN 12350-2:2011 [30]
Density of concrete mixture	EN 12350-6 [29,31]
Air content	EN 12350-7:2011 [32]
Compressive strength	EN 12390-3:2011 [33]
Absorption	PN-B-06250:1988 [34]
Depth of water penetration under pressure	EN 12390-8:2011 [35]
Depth of carbonation	prCEN/TS 12390-12:2010 [36]
Permeation of chloride ions	ASTM C 1202-05 [37]
Ordinary frost resistance	PN-B-06265:2018-10 [38]
De-icing salts frost resistance (surface scaling)	CEN/TS 12390-9:2007 [39]

**Table 6:** Physical and mechanical properties of cements.

No.	Cement Designation	Constancy of Volume (mm)	Density (g/cm^3^)	Initial Setting Time (h, min)	Specific Surface Area (cm^2^/g)	Compressive Strength (MPa)				
						2 Days	7 Days	28 Days	90 Days	360 Days
1.	C(30S-10LL)	0.5	3.00	2:15	4750	19.3	36.4	60.1	69.2	74.9
2.	C(30V-10LL)	0	2.70	2:40	4700	18.4	28.5	40.9	54.9	64.1
3.	C(35S-20LL)	0	2.96	2:45	4600	12.1	25.0	46.8	56.2	61.0
4.	C(35S-20V)	0	2.82	3:25	4350	10.7	22.8	45.4	59.1	64.8
5.	C(40V)	0	2.64	3:00	4300	15.5	26.4	36.4	49.4	61.3
6.	C(55S)	0	2.98	3:10	4330	10.5	25.7	51.5	64.2	71.5
7.	C(40LL)	1	2.92	1:55	5765	16.5	27.5	33.2	37.0	39.0

**Table 7:** Properties of concrete mixtures.

Property	Ratio w/c	Concrete Designation—Corresponding to the Composition of the Cement			
		C(30S-10LL)	C(30V-10LL)	C(35S-20LL)	C(35S-20V)
Consistency, (mm)	0.60	110	60	50	50
		S3 *	S2 *	S2 *	S2 *
	0.35	30	20	30	30
		S1 *	S1 *	S1 *	S1 *
Density, (kg/m^3^)	0.60	2360	2350	2350	2350
	0.35	2430	2420	2430	2420
Air content, (vol.%)	0.60	0.8	0.8	1.0	0.6
	0.35	1.6	1.8	2.0	2.0

* Consistency class acc. EN 12350-2:2011 [30].

**Table 8:** Results of frost-resistance tests.

Concrete Age (Days)	Tested Properties	Concrete Designation, w/c = 0.35			
		C(30S-10LL)	C(30V-10LL)	C(35S-20LL)	C(35S-20V)
28	Strength of samples after freeze-thaw cycles, (MPa)	86.7	63.4	77.2	76.4
	Strength of reference samples, (MPa)	93.8	78.5	83.7	82.7
	Decrease in strength, (%)	7.6	19.2	7.7	7.7
	Loss of sample weight after test, (wt. %)	0.0	0.1	0.1	0.1
	Scaling of the material after 56 freeze-thaw cycles in the presence of NaCl salt, (kg/m^2^)	0.12	2.77	0.89	0.55
90	Strength of samples after freeze-thaw cycles, (MPa)	94.8	71.6	84.6	85.7
	Strength of reference samples, (MPa)	95.0	84.3	85.9	84.9
	Decrease in strength, (%)	0.2	15.1	1.5	1.0
	Loss of sample weight after test, (wt. %)	0.0	0.0	0.1	0.1

**Table 9:** CO_2_ emissions from 1 m^3^ concrete and converted to 1 MPa of 28-day and 90-day concrete compressive strength.

Concrete Designation	Cement Content (kg/m^3^ Concrete)	w/c Ratio	CO_2_ Emission (kg/m^3^ Concrete)	28-Day Concrete Compressive Strength f_cm,cube 28_ (MPa)	CO_2_ Emission (kg) Converted into 1 MPa f_cm,cube 28_	90-Day Concrete Compressive Strength f_cm,cube 90_ (MPa)	CO_2_ Emission (kg) Converted into 1 MPa f_cm,cube 90_
C(30S-10LL)	300.0	0.60	136.1	42.6	3.2	50.3	2.7
C(30V-10LL)	300.0		136.1	27.9	4.9	38.3	3.6
C(35S-20LL)	300.0		102.1	33.8	3.0	39.8	2.6
C(35S-20V)	300.0		102.1	33.5	3.0	45.3	2.3
C(30S-10LL)	340.0	0.35	154.2	85.8	1.8	95.3	1.6
C(30V-10LL)	340.0		154.2	66.7	2.3	82.6	1.9
C(35S-20LL)	340.0		115.7	72.6	1.6	83.0	1.4
C(35S-20V)	340.0		115.7	70.4	1.6	82.0	1.4
